# Contribution of fish to food and nutrition security in Southern Africa: challenges and opportunities in fish production

**DOI:** 10.3389/fnut.2024.1424740

**Published:** 2024-12-04

**Authors:** Sahya Maulu, Confred Godfrey Musuka, Montshwari Molefe, Tlou Kevin Ngoepe, Ndakalimwe Naftal Gabriel, Joseph Mphande, Msumenji Phiri, Valdemiro Muhala, Messias Alfredo Macuiane, Mzime Regina Ndebele-Murisa, Oliver Jolezya Hasimuna, Thethela Bokhutlo, Mexford Mulumpwa, Victoria Ndinelago Erasmus, Wilson Jere, Chipo Hazel Dekesa, Chipo Plaxedes Mubaya, Manecas Francisco Baloi, Johannes Angala Iitembu, Enock Siankwilimba, Lin Zhang

**Affiliations:** ^1^Department of Research and Development, Centre for Innovative Approach Zambia, Lusaka, Zambia; ^2^Faculty of Science and Engineering, School of Biological and Marine Sciences, University of Plymouth, Plymouth, United Kingdom; ^3^Department of Zoology and Aquatic Sciences, School of Natural Resources, Copperbelt University, Kitwe, Zambia; ^4^Department of Aquaculture and Apiculture, Botswana Ministry of Agriculture, Selibe Phikwe, Botswana; ^5^Department of Earth and Environmental Sciences, Botswana International University of Science and Technology, Palapye, Botswana; ^6^Westville Campus, School of Life Sciences, University of KwaZulu-Nataal, Durban, South Africa; ^7^Department of Fisheries and Ocean Sciences, Faculty of Agriculture, Engineering and Natural Science, University of Namibia, Henties Bay, Namibia; ^8^Department of Fisheries, Ministry of Fisheries and Livestock, Ndola, Zambia; ^9^Department of Agriculture and Aquatic Sciences, Kapasa Makasa University, Chinsali, Zambia; ^10^Department of Aquaculture and Fisheries, Faculty of Natural Resources, Lilongwe University of Agriculture and Natural Resources, Lilongwe, Malawi; ^11^Divisão de Agricultura, Instituto Superior Politécnico de Gaza, Chókwè, Mozambique; ^12^Escola Superior de Ciências Matinhas e costeiras, Universidade Eduardo Mondlane, Quelimane Zambezia, Mozambique; ^13^Laboratório de Evolução, Universidade Federal do Pará, Bragança, Brazil; ^14^Fundo de Desenvolvimento da Economia Azul, Maputo, Mozambique; ^15^Independent Consultant, Harare, Zimbabwe; ^16^Department of Geography and Environmental Science, School of Archaeology, Geography and Environmental Science, University of Reading, Reading, United Kingdom; ^17^National Aquaculture Research and Development Centre, Department of Fisheries, Ministry of Fisheries and Livestock, Kitwe, Zambia; ^18^Department of Biological Sciences and Biotechnology, Botswana International University of Science and Technology, Palapye, Botswana; ^19^Department of Fisheries, Ministry of Natural Resources and Climate Change, Lilongwe., Malawi; ^20^Fisheries Observer Agency, Walvis Bay, Namibia; ^21^Center for Development Studies, Chinhoyi University of Technology, Chinhoyi, Zimbabwe; ^22^Faculdade de Veterinária, Universidade Eduardo Mondlane, Maputo, Mozambique; ^23^Musika Development Initiatives Zambia Limited, Lusaka, Zambia; ^24^Operations Department, Graduate School of Business, University of Zambia, Lusaka, Zambia; ^25^Freshwater Fisheries Research Center, Chinese Academy of Fishery Sciences, Wuxi, China; ^26^Wuxi Fisheries College, Nanjing Agricultural University, Wuxi, China

**Keywords:** aquaculture, blue foods, fisheries, nutrition, fish, sub-Saharan Africa

## Abstract

This study investigated the role of fish in addressing food and nutrition security challenges in Southern Africa, focusing on 10 countries including Angola, Zambia, Malawi, Mozambique, Namibia, Botswana, Zimbabwe, Lesotho, Eswatini, and South Africa. It examined the current state of food and nutrition security, fish production, and fish consumption patterns. Additionally, the study investigated the challenges and opportunities to enhance fish production in these countries thereby enhancing food and nutrition security. The findings revealed persistent challenges such as a high prevalence of food insecurity and malnutrition throughout the region. Fish production is hindered by overexploitation of fishery resources, inadequate fisheries management, susceptibility to climate-related shocks, limited investments in aquaculture, and inadequate access to input supplies such as quality fish seed and feed. Despite these obstacles, opportunities exist to promote sustainable fish production to enhance food security and nutrition. Countries endowed with extensive coastlines and inland waters exhibit significant potential for fishery development, while landlocked nations are increasingly exploring aquaculture as a viable solution. Addressing the challenges in fish production and capitalizing on opportunities requires comprehensive governance, technological innovation, policy interventions, and investment to ensure the sustainability and resilience of the fisheries and aquaculture industries in the region.

## 1 Introduction

Hunger and malnutrition are significant challenges that impact a substantial part of the global population. In 2020, about 3.1 billion people were unable to afford a healthy diet, while 2.3 billion people were considered to have moderate or severe food insecurity in 2021 (1). These challenges are exacerbated by climate risks, the contraction of global economies, conflicts, and a rapidly growing human population, making the agri-food system extremely vulnerable to shocks and disruptions (2). About a third (2.4 billion people) of the global population, of which majority are women and people living in rural areas, have no access to nutritious, safe, and sufficient food all year round (2). Due to unequal distribution of food, stunting, wasting, and overweight continue to severely affect millions of people, making it impossible for the world to meet the 2030 targets of ending hunger, food insecurity, and malnutrition in all forms (2). Additionally, the increasingly negative impact of micronutrient deficiency on the global population highlights the urgent need to transform existent food policies and focus on not only the quantity but also the quality of the food consumed (3, 88, 89).

At the same time, the global population is expanding rapidly with Africa projected to account for most of the population growth by 2050 (4). The sub-Saharan Africa (SSA) in particular, has continued to record the fastest-growing population since the 1980s (5). On the other hand, the prevalence of undernourishment remains high in Africa with the sub-Saharan region being the most affected (2). For instance, in 2020, SSA experienced the highest prevalence of undernourishment globally, with 264.2 million people affected, accounting for approximately 24.1% of the population (6, 7). Moreover, affordability for healthy diets is particularly low in sub-Saharan Africa, where an estimated 875 million people cannot afford the cost of a nutritious diet [(8, 93)]. Furthermore, the increase in the prevalence of food insecurity reached the highest in this region in 2022, while micronutrient deficiency, also known as hidden hunger, also continues to persist, affecting three-quarters of the African population (2). Therefore, it is becoming increasingly evident that traditional food production and consumption patterns are inadequate, or the nutritional value of such foods is deficient to support and nourish the projected increase in the regional population. Addressing these challenges effectively requires coordinated efforts at local, national, and international levels to ensure access to nutritious food, improve food security, and promote sustainable food systems.

Fish play a key role in the global food supply, accounting for around 17% of dietary animal protein intake, and reaching more than 50% in some countries in Asia and Africa (9). They often represent a highly affordable, easily available, and accessible source of animal protein and micronutrients that play a crucial role in food and nutrition, as well as in the income security of many people (9, 12, 90). Fish have been recognized as an important food item for providing the proteins required for human nutrition (13, 14). It is a rich source of proteins and essential fatty acids besides numerous bioavailable micronutrients required to overcome nutritional deficiency in children, expectant mothers, and adults. Expanding fish production through sustainable aquaculture and fisheries development could potentially play a significant role in improving food and nutrition security in Africa.

Despite the increasingly important role of aquatic foods in food and nutrition security, Africa’s production has remained relatively low at 7% of global production (9). Additionally, the *per capita* consumption of fish in Africa, particularly in SSA, is expected to decrease due to insufficient supply growth to match the pace of population expansion (9). Curbing the challenges of food and nutrition insecurity in the region will require increased production and consumption of nutritious foods such as fish. Regrettably, the role of fish in food and nutrition security has received less attention in Africa, which may be one of the major reasons for the consistently low fish production in this region (9). This study aimed to investigate the contribution of fish to food and nutrition security in Southern Africa. This study further explored the challenges and opportunities for increasing fish production in this region.

## 2 Methods

**Figure 1:**
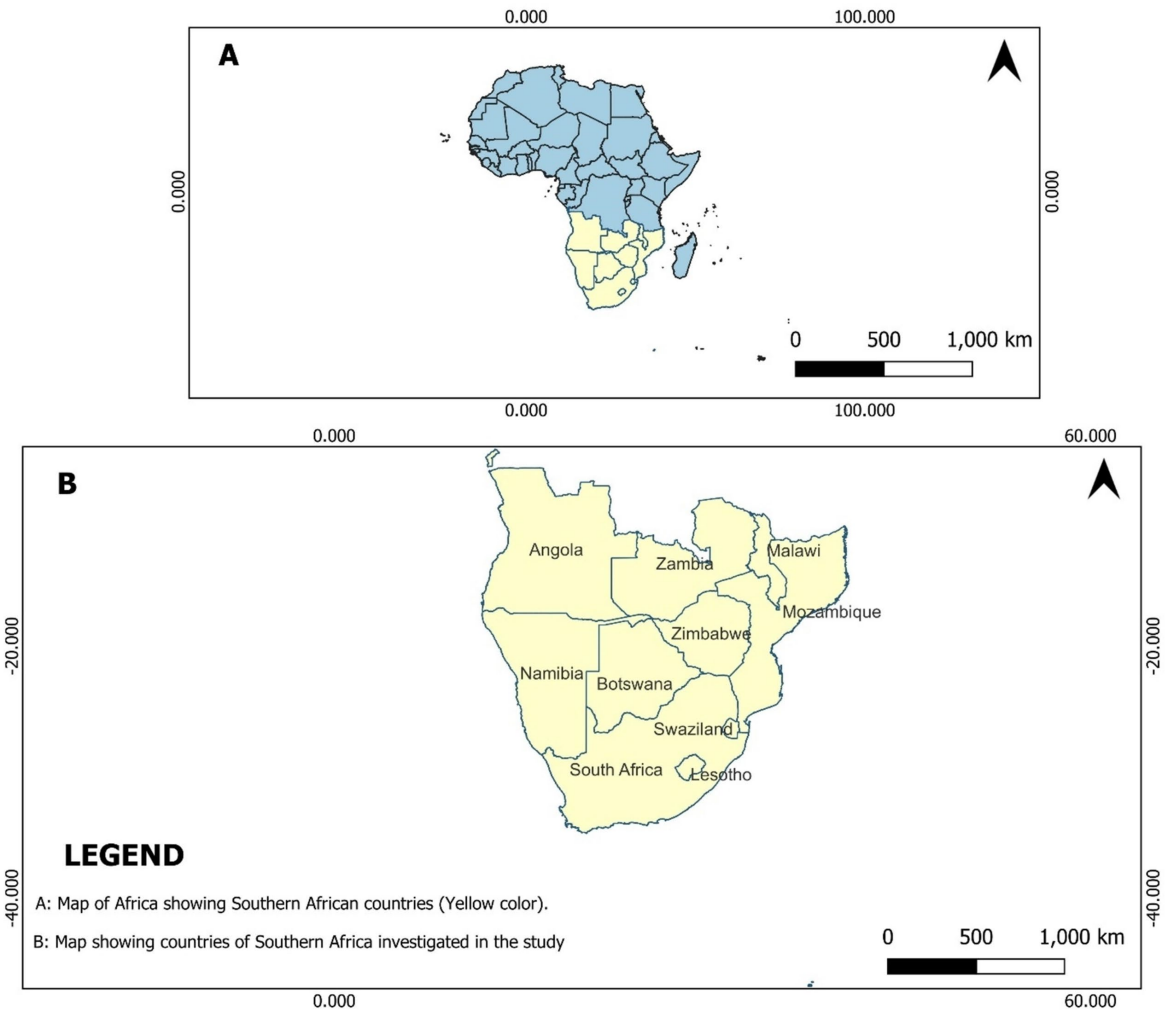
Map showing the countries in Southern Africa investigated in this study.

Ten Southern African countries belonging to the Southern African Development Community (SADC) were selected for investigation in this study (Figure 1). These countries were selected based on both their geographical characteristics and their involvement in SADC programs designed to promote fish production in the region. The study was conducted through a comprehensive review of the existing literature relating to food and nutrition security, fish consumption, and fish production in Southern African countries. The aim was to compile and analyze data sourced primarily from reputable organizations such as the United Nations Food and Agriculture Organization (FAO), World Bank, and peer-reviewed journals.

To ensure a rigorous and transparent assessment, we assembled expert teams from each country under study, with a focus on individuals with expertise in fisheries, aquaculture, or food security. By involving experts from each country, the study aimed to incorporate local knowledge and perspectives into the study. Following the formation of expert teams, independent reviews of the existing literature were conducted, with one team focusing on literature sources and the other examining FAO databases for food security indicators, nutrition data, fish production, aquatic product trade statistics, and fish consumption specific to each selected country. Upon data collection, careful comparisons were made among the various sources. In instances where minor discrepancies arose, data sourced from the FAO were prioritized to ensure transparency and consistency. Moreover, great care was taken to ensure that data comparisons between countries were based on information from the same sources and timeframes, thus ensuring uniformity in data presentation and interpretation.

Additionally, a thorough review of peer-reviewed scientific papers was conducted to identify and assess the challenges and opportunities in fish production from capture fisheries and aquaculture. This complementary information, along with the gathered data, formed the basis for a critical analysis of the obstacles facing capture fisheries and aquaculture production in the region. Moreover, it allowed the identification of opportunities aimed at promoting fish production and subsequently enhancing its role in addressing food and nutrition security concerns. This holistic approach facilitated the synthesis of relevant information to provide guidance on policies and strategies for promoting sustainable and resilient fisheries and aquaculture sectors in the region.

## 3 Results

### 3.1 Geographic and economic profile of selected countries

Key geographic, hydrological, demographic, and economic indicators for the selected Southern African countries are presented in Table 1. Angola has the largest total area, covering 1,246,700 square kilometers, and possesses substantial inland waters and coastlines. Botswana, though landlocked with no coastline, has a considerable surface area of inland waters relative to its total size. Eswatini (formerly Swaziland) and Lesotho have limited inland waters and no coastline, reflecting their small sizes. Malawi has a significant surface area of inland waters compared to its overall size. Mozambique has an extensive coastline and substantial inland waters, aligned with its large total size. Namibia’s coastline is prominent, contributing to its relatively small total size; its inland waters are noteworthy. South Africa, with its diverse geography, has an extensive coastline and substantial inland waters. Zambia and Zimbabwe, both landlocked without coastlines, have varying sizes and inland water resources. Regarding population and Gross Domestic Product (GDP) *per capita*, South Africa and Angola lead, whereas Eswatini, Lesotho, and Botswana have smaller populations and relatively higher GDP *per capita*.

**Table 1:** Key geographic, hydrological, demographic, and economic indicators of the countries studied.

Country	Total size (Km^2^)^1^	Surface area of inland waters^2^	Coastline length (km)^3^	Total renewable water (billion m^3^/year)^1^	Population (Million, 2021)^4^	GDP ($/capita, 2022)^5^
Angola	1,246,700	2,359	1,600	148.4	34.5	3256.34
Botswana	581,730	642	0	12.2	2.6	7274.23
Eswatini	17,360	70	0	4.5	1.2	4025.32
Lesotho	30,360	70	0	3.0	2.3	1107.40
Malawi	118,480	24,242	0	17.3	19.9	622.48
Mozambique	786,380	14,916	2,470	217.1	32.1	541.45
Namibia	824,290	5,101	1,572	39.9	2.5	4828.45
South Africa	1,219,090	5,509	2,798	51.4	59.4	6776.48
Zambia	752,610	14,042	0	104.8	19.5	1431.86
Zimbabwe	390,760	4,472	0	20.0	16.0	1553.54

1 FAO AQUASTAT main country database (accessed on 15 April 2024).

2 FAOSTAT land cover database (updated in 2020; CCI_LC).

3 The World Factbook, Central Intelligence Agency (CIA), United States of America (accessed on 18 April 2024).

4 United Nations, World Population Prospects (4).

5 Determined by diving total GDP (IMF World Economic Outlook Database) by population (UN World Population Prospects) in 2022.

#### 3.1.1 Population dynamics

**Figure 2:**
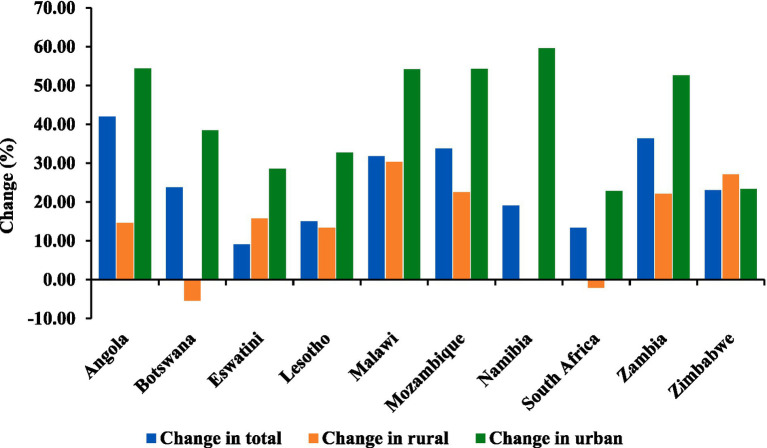
Percentage change in total population, rural population, and urban population in 2011 against 2021 by country. Data source: United Nations, World Population Prospects (5), licensed under CC BY 3.0 IGO.

Figure 2 illustrates the changes in total rural and urban populations across the 10 African countries between 2011 and 2021. Angola experienced the most significant overall population growth at 41.98%, coupled with substantial urbanization (54.42%). Botswana and South Africa showed moderate population growth (23.81 and 13.36%, respectively), with notable urbanization trends (38.46 and 22.87%, respectively). Malawi, Mozambique, and Zambia exhibited substantial population increases (31.79, 33.75, and 36.36%, respectively) and noteworthy urbanization rates (54.17, 54.32, and 52.63%, respectively). Lesotho and Eswatini saw moderate population growth (15.00 and 9.09%, respectively) and substantial urbanization (32.69 and 28.57%, respectively). Namibia and Zimbabwe experienced moderate population growth (19.05 and 23.08%, respectively) with varying urbanization rates (59.57 and 23.40%, respectively).

### 3.2 Status of food and nutrition security

**Figure 3:**
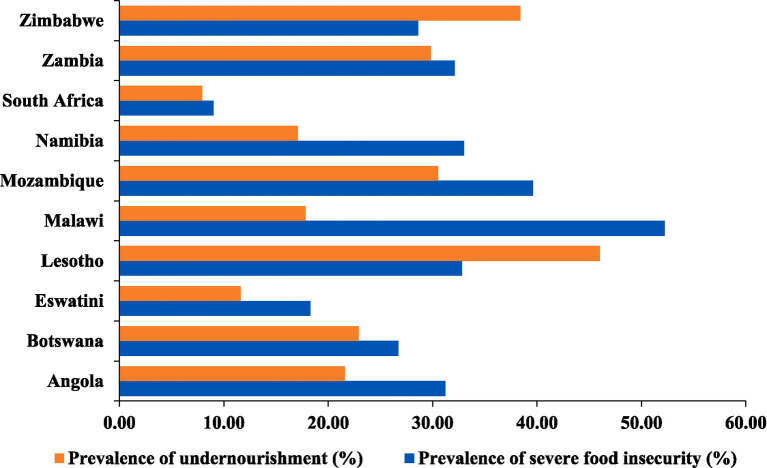
Food insecurity status by country (2020–2022, average). Data source: FAOSTAT-Suite of Food Security Indicaors (79), licensed under CC BY 4.0. Values are presented as percentages (%).

The average prevalence of severe food insecurity and undernourishment in various Southern African countries between 2020 and 2022 is depicted in Figure 3. Malawi had the highest prevalence of severe food insecurity at 52.20%, accompanied by a significant undernourishment rate of 17.8%. Lesotho also faced substantial challenges with 32.80% experiencing severe food insecurity and a high undernourishment rate of 46%. Mozambique, Angola, Zambia, and Zimbabwe exhibited notable levels of both severe food insecurity and undernourishment. Conversely, South Africa showed lower rates, with only 9.00% experiencing severe food insecurity and 7.9% facing undernourishment. Eswatini and Namibia had relatively lower prevalence rates for both indicators. Botswana had a slightly lower rate of undernourishment at 22.9%, despite facing a considerable prevalence of severe food insecurity at 26.70%.

The key nutritional security indicators in various Southern African countries are summarized in Table 2. Stunting in children under 5 years old was most prevalent in Angola, Mozambique, Malawi, and Lesotho, reflecting high levels of chronic malnutrition. Wasting, an acute form of malnutrition, was notably present in Lesotho and Mozambique. Overweight rates among young children were highest in South Africa and Botswana. The adult obesity rates were particularly notable in Angola and South Africa. Anaemia was widespread in women of reproductive age (15–45 years), with Mozambique (47.9%) and Angola (44.5%) showing the highest prevalence.

**Table 2:** Nutrition security status by country in Southern Africa.

Country	Stunting in children (<5 yrs.), 2022	Wasting in children (<5 yrs.), 2022	Overweight in children (<5 yrs.), 2022	Obesity in adults (+18 yrs.), 2016	Anaemia in women (15–45 yrs.), 2019
Angola	43.6	-	3.9	8.2	44.5
Botswana	21.6	-	10.1	18.9	32.5
Eswatini	21.2	-	7.9	16.5	30.7
Lesotho	31.8	2.1	6.9	16.6	27.9
Malawi	34.0	2.6	3.9	5.8	31.4
Mozambique	36.4	3.9	5.5	7.2	47.9
Namibia	16.8	-	5.3	17.2	25.2
South Africa	22.8	3.8	12.1	28.3	30.5
Zambia	31.4	4.2	5.4	8.1	31.5
Zimbabwe	21.6	2.9	2.7	15.5	28.9

Data source: FAOSTAT—suite of food security indicators (79).

#### 3.2.1 Cost and affordability of a healthy diet by country in Southern Africa

Table 3 provides information about the affordability and composition of healthy diets across several African countries. This reveals the cost distribution of various food categories that contribute to a balanced diet. Animal sources, vegetables, and fruits are relatively expensive components, particularly in countries such as Angola, Botswana, Eswatini, Lesotho, Malawi, and South Africa, where the cost of a healthy diet based on purchasing power parity (PPP) was relatively high. In contrast, Namibia showed a lower percentage of people unable to afford a healthy diet, while Malawi faced the highest percentage of people unable to afford a healthy diet.

**Table 3:** The estimated cost of a healthy diet (PPP dollar/person/day) in 2017.

Country	Animal source	Starchy staples	Legumes, nuts, and seeds	Vegetables and fruits	Fruits	Oils and fats	Cost of a healthy diet	% people unable to afford a healthy diet
Angola	1.01	0.84	0.40	1.20	0.72	0.17	4.33	81.4
Botswana	1.10	0.39	0.40	0.80	0.80	0.14	3.62	63.2
Eswatini	1.14	0.51	0.34	0.77	0.52	0.15	3.43	77.1
Lesotho	1.17	0.46	0.36	0.79	0.84	0.15	3.77	83.2
Malawi	1.09	0.33	0.13	0.61	0.40	0.18	2.72	94.5
Mozambique	0.99	0.41	0.21	0.84	0.39	0.19	3.03	91.2
Namibia	0.74	0.57	0.19	0.83	0.81	0.12	3.26	55.4
South Africa	1.06	0.63	0.85	0.70	0.73	0.13	4.10	65.3
Zambia	0.91	0.81	0.23	0.63	0.37	0.13	3.09	88.5
Zimbabwe	0.95	0.55	0.33	0.97	0.51	0.14	2.20	67.8

Data source: FAOSTAT suite of cost and affordability of a healthy diet (CoAHD) (6).

#### 3.2.2 Protein supply

**Figure 4:**
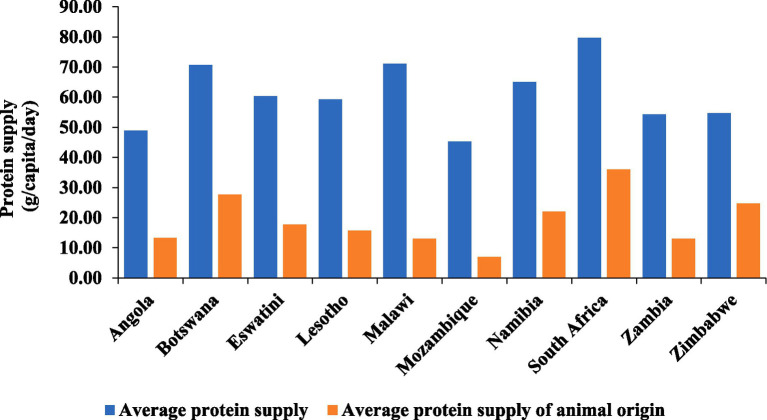
Estimated average animal protein supply by country in Southern Africa (2018–2020). Data source: FAOSTAT New Food Balances (6), (accessed on December 15, 2023), licensed under CC BY 4.0.

The average protein supply *per capita* per day and the average protein supply of animal origin for the 10 African countries are shown in Figure 4. South Africa had the highest average protein supply of 79.70 g *per capita* per day, with a substantial portion (36.00 g) originating from animal sources. Malawi and Botswana also had relatively high average protein supplies, with values of 71.00 g and 70.70 g, respectively, with a significant contribution from animal-origin protein. Mozambique had the lowest average protein supply at 45.30 g, with only 7.00 g from animal sources. Notably, the contribution of animal-origin proteins varied, with South Africa, Botswana, and Namibia showing the highest values.

### 3.3 Contribution of fish to food and nutrition

The dietary patterns related to animal protein and fish consumption across the selected countries are shown in Table 4. Angola and Mozambique showed relatively higher dietary animal protein intake (14.00 g/person/day and 7.40 g/person/day, respectively), with Mozambique having a substantial *per capita* fish share of 53.70%. On the other hand, Malawi had a high fish and seafood liking index (99.70) against Africa’s average of 75.5 coupled with significant fish consumption (23.20% *per capita* fish share). Botswana and Lesotho exhibited lower *per capita* fish shares (2.40 and 4.70%, respectively), indicating a lesser reliance on fish in their diets. South Africa stood out with the highest dietary animal protein intake (41.10 g/person/day) and a relatively low *per capita* fish share (4.40%). The fish and seafood liking index provided an interesting perspective, with Mozambique, Angola, and Malawi exceeding the African average.

**Table 4:** Contribution of fish to food and nutrition security by country in 2021.

Country	Dietary animal protein intake (g/person/day)	Fish share (%)	*Per capita* fish consumption (Kg/person/year)^1^	Fish and Seafood liking index (Africa average = 75.5; global = 100)^2^
Angola	14.00	29.80	14.20	129.00
Botswana	26.70	2.40	2.40	17.40
Eswatini	19.80	6.00	4.20	16.40
Lesotho	18.10	4.70	2.90	15.30
Malawi	12.90	23.20	10.10	99.70
Mozambique	7.40	53.70	13.90	125.20
Namibia	25.90	13.20	11.70	67.80
South Africa	41.10	4.40	6.60	32.30
Zambia	12.70	30.60	13.10	72.90
Zimbabwe	24.70	3.50	2.00	30.00

1 Data accessed from country based WAPI sheets (80).

2 Cai and Leung (81); FAOSTAT New Food Balances (accessed on December 15, 2023).

#### 3.3.1 Fish production by country

**Figure 5:**
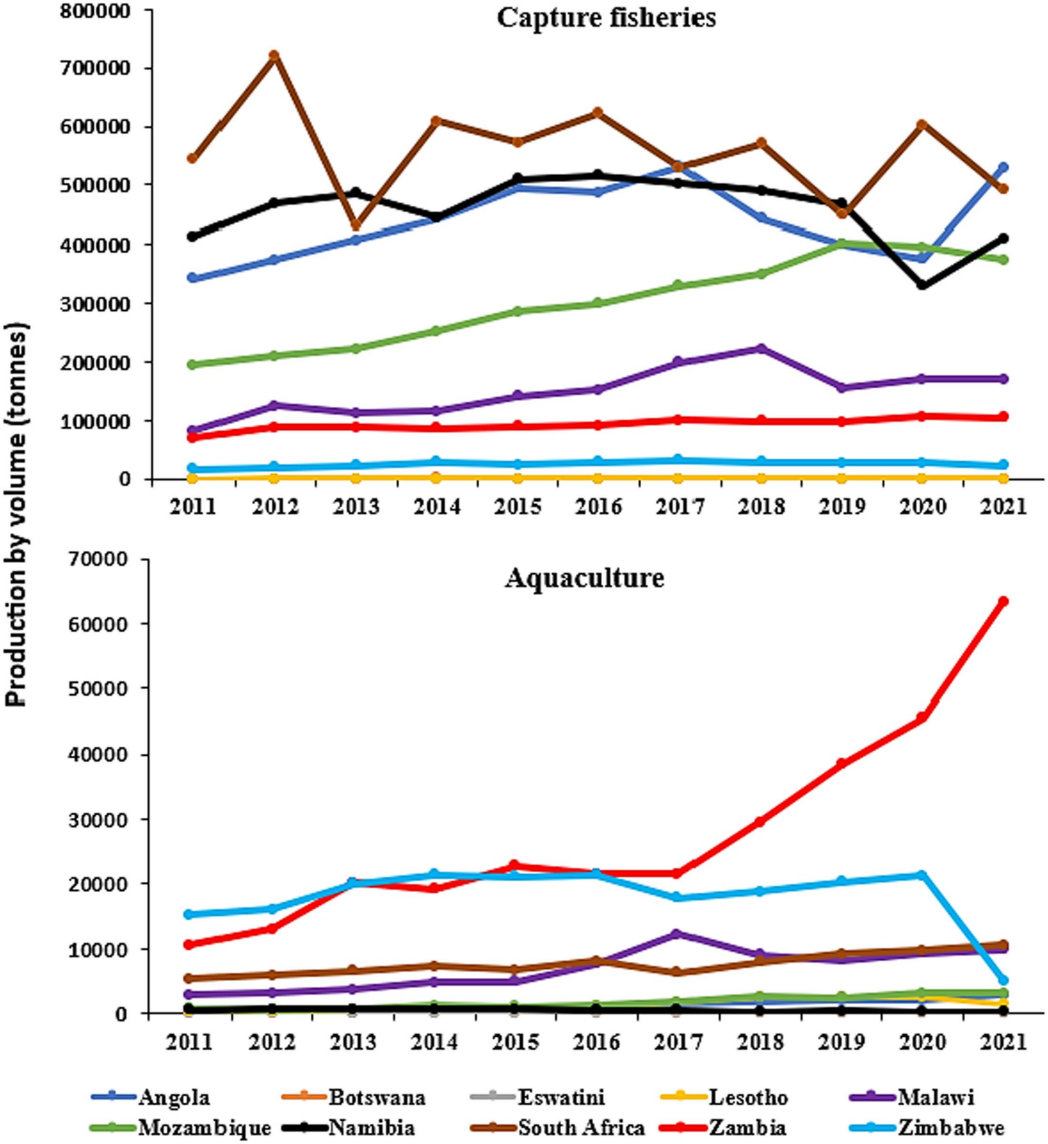
Capture fisheries and aquaculture production by country in Southern Africa, 2011–2021. Data source: FAO fishery and aquaculture statistics, global fisheries and aquaculture production (79); FAOSTAT, licensed under CC BY 4.0.

Figure 5 illustrates the quantities of fish production in tons from capture fisheries and aquaculture in the 10 Southern African countries from 2011 to 2021. South Africa consistently led in capture fisheries production, reaching 622,090 tonnes in 2016 and maintaining relatively high levels thereafter. Mozambique also showed a substantial increase, reaching 329,670 tonnes in 2020. Angola exhibited fluctuations, with a peak of 531,575 tonnes in 2017. Malawi, on the other hand, experienced a decline from 2015 to 2020. Despite variations in individual country trends, the overall total fish production from capture fisheries across these nations remained relatively stable over the 2011–2021 period, ranging from approximately 1.7 million tonnes to 2.2 million tonnes. Zambia consistently dominated aquaculture production, reaching 63,355 tonnes in (15), followed by Zimbabwe and Malawi. The results indicate an overall upward trend in aquaculture production across the region, with notable increases in Zambia. While some countries, such as Lesotho, Eswatini, and Botswana, maintain relatively low production levels.

#### 3.3.2 Aquatic products trade

**Figure 6:**
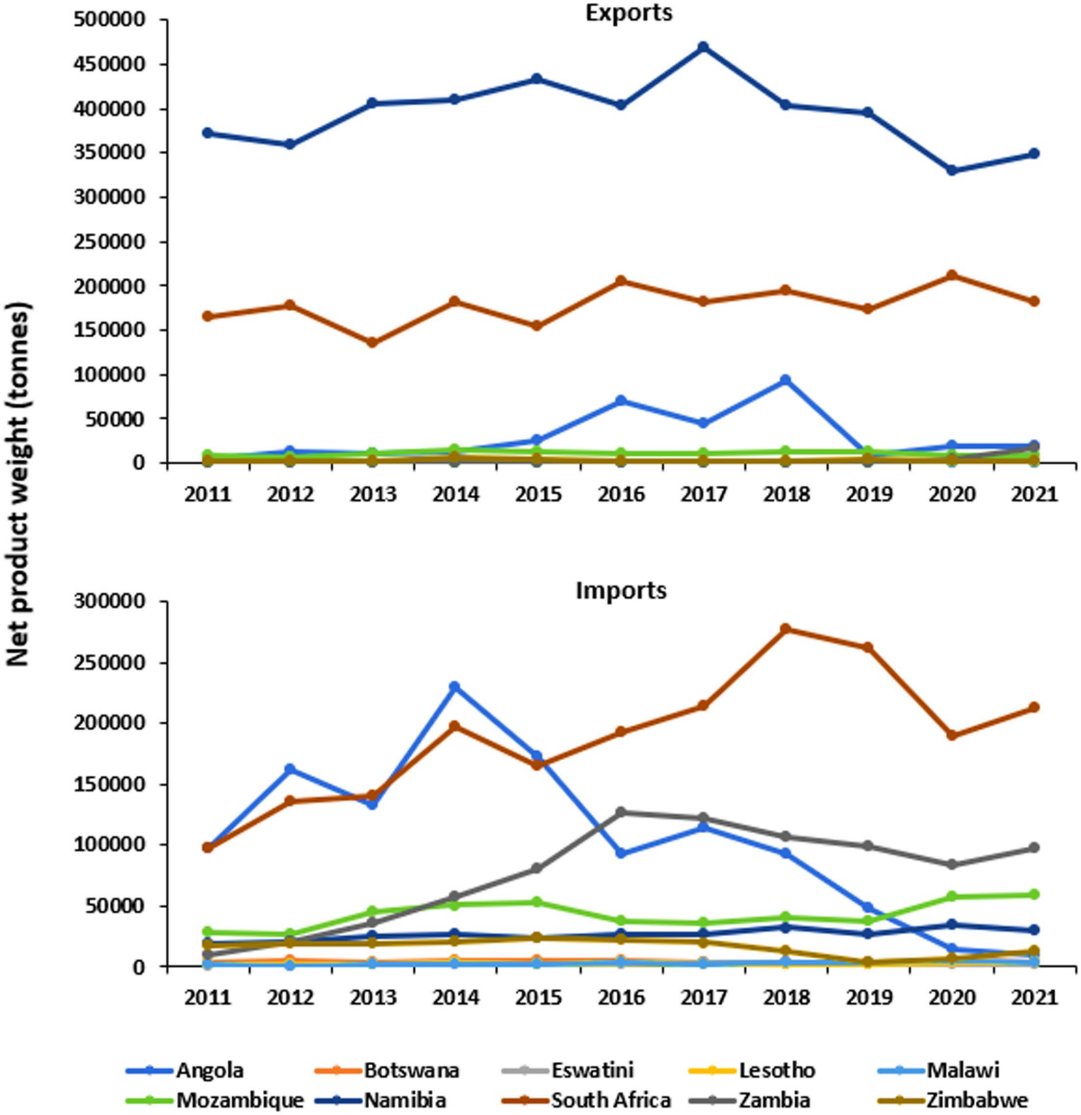
Aquatic products trade by country in Southern Africa, 2011–2021. Data Source (80); FAOSTAT, licensed under CC BY 4.0.

The volumes of aquatic products for both exports and imports (in metric tonnes) across the 10 Southern African countries from 2011 to 2021 are presented in Figure 6. South Africa experienced a notable increase in aquatic product exports, reaching a peak of 205,840 metric tonnes in 2016, before gradually declining to 182,736 metrics tonnes in 2021. Meanwhile, Mozambique consistently contributed a significant volume, ranging from 8,222 metric tonnes in 2011 to 9,250 metrics tonnes in 2021. Angola also demonstrated growth in this regard, with its aquatic product exports increasing from 5,084 metric tonnes in 2011 to 18,337 metric tonnes in 2021. Conversely, countries like Botswana and Eswatini contributed relatively small volumes, while others, including Namibia and Zambia, showed fluctuations.

The volume of aquatic product imports varied across countries during the same period. Angola maintained a high volume of imports throughout the period, peaking at 229,849 metric tonnes in 2014 and declining to 9,885 metric tonnes in 2021. Mozambique also exhibited substantial imports, with the highest volume of 59,112 metric tonnes recorded in 2021. South Africa consistently imported a significant amount of aquatic products, peaking at 276,770 metric tonnes in 2018. By contrast, countries such as Botswana, Eswatini, Lesotho, and Zimbabwe generally had lower import volumes in the region. Overall, the regional total import volume fluctuated over the years, reaching a peak of 597,051 metric tonnes in 2014, and slightly decreasing to 436,222 metric tonnes in 2021, to which Angola’s large drop in its import are largely noticeable.

In general, the data indicate a trend of decreasing exports, coupled with increasing imports for most of the countries in Southern Africa.

## 4 Discussion

### 4.1 Food and nutrition security status

The pursuit of global food stability is a complex endeavor influenced by a diverse array of factors impacting production, supply, and consumption dynamics (16). Several factors, such as population growth, *per capita* income growth, inflation, and urbanization, strongly influence food demand [(17, 18, 90)]. In the Southern African region, the population is growing rapidly, with more than a 20% growth rate recorded between 2011 and 2021 in the selected countries except for Namibia, Lesotho, South Africa, and Eswatini. During the same period, higher growth rates were observed in urbanization than in the total population or rural population across the countries. The results suggest a general trend toward urbanization across the countries, reflecting ongoing demographic shifts and the associated challenges and opportunities for urban development and resource allocation. The current study further revealed that countries with higher urban populations also had higher GDP, suggesting higher *per capita* income growth, which is likely to increase the food demand especially for nutritious diets in the region.

A significant proportion of the population across the studied countries is affected by food and nutrition insecurity. Except for South Africa and Eswatini, the prevalence of severe food insecurity surpassed the African average by 23.4% between 2020 and 2022. Similarly, the incidence of undernourishment exceeded the African average of 19.27%, except for South Africa, Namibia, Malawi, and Eswatini. Notably, Malawi had the highest prevalence of severe food insecurity, affecting over 50% of its population, whereas Lesotho exhibited the highest incidence of undernourishment, affecting nearly half of its population. While many challenges in the region are similar, the degree of impact varies across countries, revealing differing levels of vulnerability to food insecurity. For example, in countries such as Malawi and Mozambique, where the prevalence of food insecurity was the highest, climate shocks have exerted a significant adverse impact on rain-fed agricultural systems and fish production (19, 20). An estimated 800,000 hectares of standing crops have been destroyed by cyclones and related floods that occurred in 2019 in Mozambique, Malawi, and Zimbabwe, affecting approximately 3.2 million people in these countries (21). A study conducted by Muhala et al. (22) in Mozambique revealed that cyclones Kenneth and Idai, which struck in 2019, resulted in the destruction of approximately 2,457 fishing vessels and equipment, 169 fishponds, 206 cages, 58 fish tanks, and fish seed stocks totaling 863,500 units. Additionally, many countries in the region depend heavily on rain-fed agriculture, making them particularly susceptible to the impacts of climate change.

Nutritional challenges are also highly prevalent in Southern Africa, including high rates of stunted growth in children and anemia among women of reproductive age. Despite progress in maternal, infant, and young child nutrition (MIYCN) across the region, reducing anemia prevalence among women of reproductive age remains a challenge (1). A key contributing factor to these challenges is likely low dietary diversity, where diets primarily consist of carbohydrate-based meals with minimal consumption of protein-rich foods of animal origin. In Malawi for instance, most people primarily consume cereals, grains, and legumes, whereas protein-rich foods such as fish, red meat, and eggs are consumed sparingly (23). Mozambique exhibits inadequate dietary quality, largely reliant on staple foods, leading to a deficiency in essential micronutrients, thereby exacerbating the prevalence of stunting in children (24). Heavy reliance on starchy staples leads to significant disparities in the consumption of healthy food groups, with variations in magnitude influenced by factors like household location and economic status (25). This challenge is often compounded by poor nutrition governance and high poverty levels, which hinders people’s ability to provide affordable and accessible dietary diversity. For example, Eswatini has moderate nutrition governance through initiatives like micronutrient supplementation and malnutrition treatment, but lacks national dietary guidelines, limiting practitioners’ ability to offer tailored advice (26).

Poverty is a major hindrance to food access in most countries in the region, evident in the alarmingly high percentages of the population living below the poverty datum line (92). Poverty affects access to not only quantity but also quality foods. The onset of the COVID-19 pandemic further exacerbated poverty across many nations, with greater impacts experienced in developing countries (27, 28). As a result, food shortages are becoming even more prevalent in many countries in Southern Africa. Additionally, social conditions, such as health, nutrition, education, and housing, influence productivity, thus affecting the poverty status of communities. In turn, social conditions are influenced by poverty, affecting households’ ability to improve their productivity (9). Improving food security and nutrition in the region requires comprehensive efforts to enhance resilience in food production, promote inclusive policies, and improve economic conditions.

### 4.2 Nutritional and social benefits of fish consumption

Sufficient protein consumption is crucial for overall human health and growth (29). Animal source foods (ASFs) are considered superior for human nutrition and health due to their well-balanced amino acid profile and ease of digestion compared to other protein sources. In contrast to plant-based foods, ASFs provide larger amounts of superior-quality proteins, along with enhanced bioavailability of vitamin A, vitamin D3, iron, iodine, zinc, calcium, folic acid, and essential fatty acids (30). The World Health Organization (WHO) identifies ASFs as the optimal source of nutrient-dense nourishment, especially for children between the ages of 6 and 23 months (31). However, ASFs stand out as the most expensive food items consumed in Southern Africa, with over 50% of the population across countries unable to afford a nutritious diet. Of note were the alarming rates observed in Malawi and Mozambique, where over 90% of the population faced this challenge, coinciding with the countries experiencing the highest levels of poverty. As cost and affordability are significant barriers hindering consumers’ access to nutritious foods (32), it is imperative to address cost issues to encourage the consumption of ASFs within the Southern African region.

Fish, being a relatively cheaper protein source, contributes significantly to nutrition security beyond providing calories and proteins (11). It is also a source of essential fatty acids, particularly omega-3 polyunsaturated fatty acids (PUFAs) like docosahexaenoic acid (DHA) and eicosapentaenoic acid (EPA) which promote brain development, cognitive function, and overall growth (10). Small pelagic fish such as *Engraulicypris sardella* (locally known as Usipa/bonya) common in Malawi and *Limnothrissa miodon* (Kapenta) found in Zimbabwe and Zambia, stand out as some of the most affordable options and are also exceptionally rich in long-chain polyunsaturated fatty acids (LC-PUFAs) (11, 33). Therefore, promoting fish consumption can help address nutritional challenges in the Southern African region.

Additionally, fish are a source of essential micronutrients, including vitamins D, A, and B (with B12 being associated with seafood), as well as minerals, such as calcium, phosphorus, iodine, zinc, iron, and selenium (14, 90). In pregnant women, vitamin D deficiency is associated with an increased risk of preeclampsia, gestational diabetes, preterm birth, and low birth weight (34). Vitamin A deficiency, a leading cause of preventable childhood blindness, weakens the immune system and contributes to anemia, while vitamin B is crucial for preventing anemia as well as neurological and cognitive issues (34, 90). Fish can help address iron deficiency, which leads to anaemia, a widespread problem in women of reproductive age in Southern Africa. Zinc, which is also highly available in fish, is vital for growth, development, and immune function, and its deficiency is linked to the prevalence of child stunting (89). Therefore, pregnant, and lactating women, infants born through breast milk, young children, and those vulnerable to malnutrition can benefit from fish consumption, thereby reducing maternal and child mortality risks.

In a comprehensive analysis pooling prospective studies and randomized clinical trials, Mozaffarian and Rimm (35) associated fish consumption with a 36% reduction in heart disease and heart attacks and a 12% reduction in overall mortality. Fish offer a comprehensive nutritional package with potential health benefits across various populations and age groups (34). Despite variations in nutritional content (e.g., fatty acid profile) among fish species owing to factors such as seasonality, habitat, trophic level, and diet (34, 36, 37), fish remain a nutritious food item with the potential to curb hunger and nutritional challenges in developing countries. Recognizing the nutritional value of fish is crucial for advocating diverse and balanced diets in the region. However, formulating targeted nutritional policies and interventions to address food insecurity and nutritional deficiencies requires a better understanding of variations in fish consumption across the region.

Furthermore, there is a need to recognize the multifaceted contribution of fish to food and nutrition security, beyond direct consumption. Fish also indirectly contribute to food and nutrition security through income generation, although this pathway is often neglected when assessing the contribution of fish (14, 38, 39). The income generated from fish production allows households to enhance their purchasing power for additional nutritious food (34, 40, 41). Fish creates employment opportunities, particularly for women involved in various stages of the value chain, including the production, processing, and trading of fish and fish products.

In Southern Africa, fisheries and aquaculture provide employment for an estimated 3.3 million individuals, constituting approximately 1% of the total population (42). As the demand for fish continues to escalate, it will be necessary to boost fish production, consequently expanding employment opportunities and granting employed households the opportunity to enhance their food and nutrition security capacity. Nevertheless, the multitude of pathways through which fish contribute to food security and nutrition complicates the quantification of their overall impact (34). Thus, the empirical link between fish production and food security warrants further investigation, considering various pathways beyond direct fish consumption.

### 4.3 Fish consumption in Southern African

Fish consumption in the Southern African region varies across countries mainly due to factors such as availability, accessibility, and affordability. In countries like Angola, Mozambique, Zambia, Malawi, and Namibia, *per capita* fish consumption exceeded the regional averages of 8.3 kg/person/year for sub-Saharan Africa and 10.0 kg/person/year for Africa in 2019. Fish contributes a significant share of the dietary animal protein consumed in these countries, aligning with higher liking indices for fish and seafood. In Angola, fish provide approximately 30% of the population’s animal protein intake despite persistent nutritional challenges in rural areas due to poor access to fish products (9). Mozambique relies on fisheries for income and sustenance, with fish accounting for approximately 53% of the nation’s total animal protein consumption. Similarly, Zambia benefits around 30% of the national dietary animal protein from fish consumption and the *per capita* fish consumption is projected to rise by 2030 (43). In Malawi, approximately 23% of the animal dietary protein consumed is supplied by fish (more especially the small fish species, i.e., *E. sardella*) despite reported variations across different socio-economic status groups and proximity to fishing areas (44). Namibia derives a relatively modest (13.20%) of its animal dietary protein from fish, but there is potential for this figure to rise, even though certain regions within the country encounter difficulties in both accessing and affording fish products (45). Surprisingly, these countries, except for Namibia also exhibit higher prevalence rates of stunting in children and anaemia in women of reproductive age on average compared to countries with lower reliance on fish for dietary protein in the region. This highlight the importance of sufficient variety in diet, the opposite of which is widely recognized as a key factor in determining stunted growth in children (46, 47).

Troell et al. (37) also noted that the overall benefits of integrating fish into one’s diet depend largely on the balance of other dietary components and whether essential macronutrients and micronutrients are already being adequately consumed to meet daily requirements. A study conducted in Zambia’s most fish-consuming region observed that despite high consumption rates of fish, stunting in children persisted, mainly due to low dietary diversity (48). Promoting the consumption of a variety of foods, including vegetables, fruits, and animal products, in early childhood can enhance growth and development (49).

Marinda et al. (50) associated stunting in children with the quality of fish consumed which highlights the significance of producing and consuming quality fish. Several factors can compromise the quality of fish consumed, including post-harvest losses, environmental factors, and contamination by pollutants, such as heavy metals and pesticides (11, 51, 52). In Lesotho, fish consumption patterns are influenced by availability and affordability of widely accessible imported canned and frozen fish products (42, 53). Similarly, fish consumption in Malawi is influenced by proximity to fishing areas (44). Disparities in national fish consumption, with coastal communities consuming higher quantities in their diets, have been noted in several countries, including South Africa, Namibia, and Angola (45, 54, 55). Thus, fish consumption patterns across the region are predominantly shaped by accessibility and affordability, underscoring the necessity of national and regional initiatives to guarantee accessibility to fish products by all groups of society.

The current study further revealed a general trend of increasing imports and decreasing exports of aquatic products during the 2011–2021 period, reflecting the increasing demand for fish products resulting from a growing population. The data also highlight the importance of aquatic product imports in meeting national fish demand in the region where regional markets play an increasingly important role (42). A slight decline in both categories was observed after 2018 for most countries, which is likely due to the COVID-19 pandemic that restricted fish trade not only in the Southern African region but also in the global market. Therefore, there is a need to strengthen food systems using resilience measures to ensure the uninterrupted provision of nutritious foods in the face of global pandemics and environmental shocks. In fishery-dependent communities, ineffective strategies for diversifying food sources exacerbate the overexploitation of wild fishery resources, leading to detrimental effects on both current and future fish consumption patterns (9). Intentional policies that encourage and maintain the diversification of food sources other than fish products will supplement and maintain the production, distribution, utilization, and ultimately the consumption of nutritious foods in the region.

### 4.4 Overview of fish production in the region

With the global population expected to surpass 9 billion by 2050 (4), the paramount concern revolves around the impact of this growth on food security dimensions, including availability, accessibility, utilization, and stability. Particularly noteworthy is the projected population rise in Sub-Saharan Africa, a region that is already struggling with high levels of food insecurity and malnutrition. Hence, it is imperative to carefully examine the future of fish production, discerning its potential to substantially alleviate and mitigate these complex challenges.

#### 4.4.1 Capture fisheries

Fish production in the Southern African region is primarily driven by capture fisheries, in line with broader continental trends (13, 82). Challenges, such as overexploitation in capture fisheries, persist, highlighting the importance of sustainable management practices. While capture fishery production has shown relative stability across countries over the past decade, the increasing demand for aquatic products cannot be sustained solely by reliance on capture fisheries. This is particularly significant as populations have grown in all the countries during the same period. Notably, Mozambique has experienced notable growth, signalling its emergence as a significant player in the regional fisheries sector. The country boasts extensive fishery resources and a thriving fishery sector, with significant potential for growth in both domestic and foreign markets (42). Similarly, Angola’s fisheries sector continues to be vital for the national economy despite facing challenges such as overfishing and hydrological changes (55). Malawi’s capture fisheries sector, encompassing both small-scale and large-scale operations, contributes to over 90% of the nation’s fish production, with Lake Malawi alone accounting for more than 90% of the total catch (56). Recent increases in fish catches have been observed in Malawi, but their implications remain unclear due to concurrent changes in species composition (57). Zambia’s abundant freshwater resources, spanning approximately 15 million hectares, offer significant potential for the development of its capture fisheries sector. Nevertheless, the industry has faced challenges, such as overexploitation and the impacts of climate change, resulting in stagnant production in recent decades (43). Botswana faces challenges in monitoring and reporting fish production, leading to inaccurate current estimates. For instance, production from major fishery resources in the country, such as the Okavango Delta, has remained unreported since 2017, highlighting the need for improved data-collection mechanisms (58). Away from the seashores that Mozambique, Namibia, and Angola comparatively and competitively enjoy, Zambia, Zimbabwe, and Botswana, share the benefits of the Zambezi River that enhance fish production and trade on their borderline corridors.

Lesotho’s capture fishery relies largely on hook and line fishing, with ongoing efforts to expand aquaculture production, promising growth for the sector, albeit with management challenges. Zimbabwe fishery sector primarily includes commercial, artisanal, and recreational fishing operations focused on lakes, particularly Lake Kariba, and is complemented by smaller water bodies for subsistence fishing (59, 84). Namibia’s fishing industry benefits from the productive Benguela Current System, although challenges such as pilchard depletion persist, and efforts to increase marine aquaculture production align with long-term sustainability goals. Besides, Namibia boasts of a highly commercialized and well-managed fishing industry in the region, contributing significantly to national and regional fish production (45, 60). Lesotho and Eswatini have minimal fisheries development due to their limited water resources, primarily consisting of rivers and small reservoirs, which are suitable mainly for subsistence fishing activities (42). South Africa’s extensive capture fisheries sector includes commercial, recreational, and subsistence fishing, with the commercial segment comprising highly industrialized offshore fisheries and more traditional near-shore fisheries with lower capital requirements (55). The observed fluctuations in capture fishery production in the region reflect the complex interplay of environmental factors, fishing practices, and management strategies. Understanding these trends is essential for sustainable fisheries management, ensuring food security, and addressing economic implications within the region.

#### 4.4.2 Aquaculture

Aquaculture across the Southern African region remains in its infancy stages, with varying degrees of development and challenges across countries. However, Zambia’s aquaculture production, ranked 5^th^ in Sub-Saharan Africa and leading in Southern Africa, is experiencing significant growth and is soon to outpace capture fisheries production, driven by strong government support, private investments in cage aquaculture, and the abundance of water resources (61, 62, 91). Zimbabwe, ranked among the top 10 aquaculture producers in sub-Saharan Africa, shows promise for further expansion due to growing support from both government and donor-funded initiatives (63). Similarly, Botswana’s growing aquaculture sector exhibits significant potential for future expansion, propelled by rising investments, primarily from the private sector. Malawi’s aquaculture production remains modest, accounting for approximately 5% of the nation’s total fish production (57). However, governmental and support agencies are increasingly acknowledging their potential. In Angola, aquaculture production is predominantly small-scale, centered on communal ponds for local consumption; nevertheless, there is a discernible increase in medium to large-scale commercial operations (54). Additionally, ongoing efforts are underway to promote shrimp aquaculture production through sustainable resource exploitation.

Eswatini’s aquaculture sector is currently underdeveloped, but there is potential for increasing tilapia production, contingent upon the formulation of a national aquaculture strategy to guide sectoral growth (90). Aquaculture in Mozambique holds vast untapped potential to become a key economic driver, despite facing challenges such as infrastructure deficiencies, limited access to inputs, and low yields (22). Aquaculture in South Africa comprises freshwater and marine sectors, with freshwater limited by the water supply and marine experiencing rapid growth, particularly focusing on high-value species (64, 65). The overall upward trajectory in aquaculture production in the region suggests growing recognition of the importance of aquaculture in meeting the demand for fish products, improving food security, and contributing to economic development. The statistics also highlight the variability in aquaculture development among these nations, which is influenced by factors such as investment, infrastructure, and government policies. Sustainable management practices and collaboration between countries could further enhance the positive impact of aquaculture on regional economies and food systems.

#### 4.4.3 Challenges

The Southern African countries face multifaceted challenges in their fisheries and aquaculture sectors. These challenges range from overfishing, infrastructural limitations, and outdated legislation to environmental impacts such as climate change. Overfishing, driven mainly by a growing population, is a major challenge leading to stagnated production in capture fisheries in many countries in the region, including Zambia, Zimbabwe, Malawi, Namibia, and Angola. Emerging challenges such as climate change could significantly affect fish populations, distribution, and productivity, although the extent of its effects is not clearly understood due to lack of quantification. The decline in aquaculture production in Mozambique, Malawi, and Zimbabwe has been attributed to climate-associated events such as cyclones, droughts, and flooding (20, 57). Furthermore, these events are widespread in several Southern African countries, including Eswatini, Mozambique, and South Africa, with varying impacts on fish production (66, 67). These challenges are likely to exacerbate the mismanagement of fishery resources (68). Additionally, a mounting challenge arises from invasive species in many key fishery resources, particularly in shared water bodies like Lake Kariba and the Zambezi River basins (69). This threat negatively impacts capture fisheries production, biodiversity, and aquaculture breeding programs, particularly due to the risk of interspecific hybridization with indigenous species.

Consequently, deficiencies in essential micronutrients and fatty acids are likely to worsen with declining capture fisheries production, considering that the sector is the major supplier of fish food in Southern Africa. Management measures are present in all countries except Lesotho and Eswatini; however, enforcement is weak across countries. Addressing these challenges requires comprehensive governance, and technological changes to ensure the continued contribution of capture fisheries to food security. For instance, inadequate port facilities, postharvest losses, and high operational costs in aquaculture hinder fish production growth in Angola, whereas fragmented extension services, climate change, and governance issues impede sectoral development in Botswana [(70, 71)].

Malawi encounters various management challenges, such as overexploitation, over-capitalization, illegal, unreported and unregulated (IUU) fishing, conflicts with industrial fisheries, and post-harvest fish losses (56, 72). Mozambique’s challenges include a lack of skilled personnel and damage from natural disasters (20), while Zambia struggles with overexploitation and regulatory conflicts in fisheries and limited access to capital and expertise in aquaculture. Zimbabwe’s fisheries and aquaculture sectors suffer from the absence of a comprehensive policy framework and various operational challenges, including input shortages and infrastructure deficiencies (63). Lesotho face challenges such as a shortage of trained personnel and limited funding, whereas Namibia contends with external factors such as climate change, volatile fuel prices and conflicts with mining activities that mainly affect capture fisheries (73). In South Africa, overexploitation and environmental concerns threaten marine resources, while regulatory complexities and economic hurdles constrain aquaculture expansion. Eswatini faces challenges, such as limited fisheries and aquaculture development, mainly due to limited access to water resources, leading to low investments in the sector (SADC, 2021).

Major challenges facing the aquaculture industry in the region are generally the same and include the high cost of quality fish feeds, poor quality fish seed, and low investment in the industry. In addition, fish diseases are emerging in the region and will have adverse effects on fish production as aquaculture production expands (57). These challenges are further exacerbated by the lack of appropriate technological advancements and inadequate research, coupled with inadequate government support in research and development. Moreover, aquaculture is poorly integrated with other food production systems to enhance productivity. Despite the potential benefits of integrating aquaculture with crop and livestock production, there is limited investment in the sector, compounded by a lack of development infrastructure, credit lines, information, training, and expertise across the region. Notwithstanding the favorable climate in the region for cultivating potential aquaculture species, limited progress has been made in this direction.

#### 4.4.4 Opportunities

Despite the challenges highlighted above, opportunities to promote fish production from fisheries and aquaculture exists across the region. Countries such as Angola, Mozambique, South Africa, and Namibia boast extensive coastline lengths and inland water surface areas, providing abundant opportunities for marine and freshwater fisheries. These resources, coupled with substantial renewable water reserves, contribute to the robust fishery sectors and support food security initiatives through the availability of diverse aquatic resources. For instance, Mozambique’s diverse natural environments and comprehensive legal frameworks support both marine and inland fisheries, with aquaculture playing an increasingly significant role. Angola’s rich marine ecosystem and coastal resources present vast opportunities for fisheries development (55). South Africa’s fishing and aquaculture sectors, although relatively small in GDP contribution, hold the potential for expansion and sustainability through initiatives promoting sustainable practices and species diversification. Furthermore, Namibia, benefiting from productive fishing grounds and effective management systems, aims to increase aquaculture production and enhance export earnings through the strategic initiatives outlined in its Vision 2030 and Master Plan for Aquaculture (FAO, 2024). In its nascent phase, Namibia’s mariculture sector holds promise for making a substantial contribution to food security (74). Conversely, landlocked countries, such as Botswana, Eswatini, Lesotho, Malawi, Zambia, and Zimbabwe, have limited access to coastal or marine water bodies, impacting their fishery potential. However, efforts to harness alternative water sources and promote sustainable aquaculture practices can contribute to food production and livelihoods in these regions. Botswana’s ephemeral water bodies (i.e., dams, swamps, and floodplains) and main rivers offer favorable environments for fish productivity (75, 76), whereas Malawi, despite persistent climate-related challenges, has the potential to expand aquaculture production owing to its abundant land and water resources. Kaimila et al. (77) estimated that 15–20% of the land in Malawi is suitable for aquaculture production besides underutilized for cage aquaculture on Lake Malawi. With its newly launched fisheries and aquaculture policy, Zambia is promoting aquaculture through various projects and initiatives to enhance production and improve food security and livelihoods. Zimbabwe, facing a significant deficit between fish demand and supply, highlights opportunities for aquaculture expansion supported by abundant land and water resources and government commitment, while Eswatini aims to tap into its favorable climate for tilapia cultivation through strategic planning and support from organizations such as the FAO (67). Lesotho’s aquaculture industry, although currently small in scale, possesses notable economic significance, primarily attributable to the cultivation of high-value species, such as trout (65). Moreover, the geographic features of Zimbabwe and Lesotho offer two distinct prospects for fish farming: highlands with warm water temperatures exceeding 24°C, ideal for cultivating warm water species such as tilapia, and lowlands with cooler temperatures providing suitable conditions for rearing high-value cold-water species such as trout (53, 78).

Given the unlikely prospect of increased capture fisheries in keeping pace with population growth, aquaculture emerges as the primary avenue for sustainably boosting fish production in the region. This acknowledgment is widespread among governments and is evident in the recognition and prioritization of aquaculture initiatives. Moreover, the Southern African Development Community (SADC) has developed a regional aquaculture strategy and action plan (RASAP) that envisions the region as a leader of sustainably produced aquaculture products in Africa to contribute significantly to food security, employment, poverty alleviation, and economic growth across the region by 2025 (85). However, achieving this will require fostering science and technology sharing, strengthen research collaborations, and establish conducive environments for both public and private investments across key industries, such as fish seed production, feed technology, fish processing, and trade.

## 5 Conclusion

This study investigated the contribution of fish to food and nutrition security in Southern Africa and highlighted the challenges and opportunities in fish production. The study revealed that severe food insecurity and high prevalence rates of stunting in children and anaemia in women of reproductive age remain major challenges affecting the region. Fish consumption has emerged as a critical component for addressing these challenges, offering a rich source of essential nutrients and potential economic benefits through aquaculture and fisheries. However, disparities in access, affordability, and dietary diversity persist across the region, necessitating targeted interventions to promote equitable access to nutritious foods including fish products. Furthermore, the Southern African region offers diverse opportunities for advancing fisheries and aquaculture to contribute to economic development, poverty alleviation, and food security. Understanding and leveraging the diverse hydrological landscapes across the region is crucial for optimizing food and fish production, ensuring resilience in the face of environmental challenges, and advancing sustainable development goals. However, the potential of capture fisheries and aquaculture to enhance food security and contribute to macro-economic development hinges on effective governance mechanisms and tailored policy interventions, which vary across countries.
